# A hybrid strain and thermal energy harvester based on an infra-red sensitive Er^3+^ modified poly(vinylidene fluoride) ferroelectret structure

**DOI:** 10.1038/s41598-017-16822-3

**Published:** 2017-12-01

**Authors:** Sujoy Kumar Ghosh, Mengying Xie, Christopher Rhys Bowen, Philip R. Davies, David J. Morgan, Dipankar Mandal

**Affiliations:** 1 Organic Nano-Piezoelectric Device Laboratory (ONPDL), Department of Physics, Jadavpur University, Kolkata, 700032 India; 2 Institute of Nano Science and Technology, Phase-10, Sector-64, Mohali, 160062 India; 3 Department of Mechanical Engineering, University of Bath, Bath, BA2 7AY UK; 4 Cardiff Catalysis Institute, School of Chemistry, Cardiff University, Cardiff, CF10 3AT United Kingdom

## Abstract

In this paper, a novel infra-red (IR) sensitive Er^3+^ modified poly(vinylidene fluoride) (PVDF) (Er-PVDF) film is developed for converting both mechanical and thermal energies into useful electrical power. The addition of Er^3+^ to PVDF is shown to improve piezoelectric properties due to the formation of a self-polarized ferroelectric β-phase and the creation of an electret-like porous structure. In addition, we demonstrate that Er^3+^ acts to enhance heat transfer into the Er-PVDF film due to its excellent infrared absorbance, which, leads to rapid and large temperature fluctuations and improved pyroelectric energy transformation. We demonstrate the potential of this novel material for mechanical energy harvesting by creating a durable ferroelectret energy harvester/nanogenerator (FTNG). The high thermal stability of the β-phase enables the FTNG to harvest large temperature fluctuations (ΔT ~ 24 K). Moreover, the superior mechanosensitivity, S_M_ ~ 3.4 VPa^−1^ of the FTNG enables the design of a wearable self-powered health-care monitoring system by human-machine integration. The combination of rare-earth ion, Er^3+^ with the ferroelectricity of PVDF provides a new and robust approach for delivering smart materials and structures for self-powered wireless technologies, sensors and Internet of Things (IoT) devices.

## Introduction

There is a widely recognized need for the development of sustainable energy sources for our modern society due to the depletion of fossil energy resources and their impact on the world atmosphere. The miniaturization of commercial wearable device systems, wireless sensors and small electronic systems has reduced their power requirement to the mW and even µW level. In this regard, the conversion of fluctuating mechanical and thermal energies into electrical energy by exploiting piezoelectric and pyroelectric materials has attracted considerable attention^1–6^. Despite the high piezoelectric activity and properties of several inorganic and semiconducting piezoelectric materials, their toxicity (many contain lead for example), high processing temperatures, complex fabrication steps, and their intrinsic rigid and brittle mechanical properties restrict some of their practical applications^7^. In contrast, piezoelectric polymers such as poly(vinylidene fluoride) (PVDF) and its co-polymers exhibit a number of attractive properties such as, flexibility, light weight, chemical resistivity, large area processing feasibility, low processing temperature and environmental compatibility (lead free). In recent years, it has been noticed that PVDF is one of the most studied engineering polymers due to its ferro-, piezo- and pyro-electric properties^8^. The material is a semi-crystalline polymer that exhibits primarily four crystalline polymorphs; namely α-, β-, γ-, and δ-phases. Among them, the β-, γ-, and δ-phases are electro-active. In particular, β-phase (all trans, *i*.*e*., TTTT conformation) is fully polar and ferroelectric, as the polarization can be repeatedly switched between opposite but energetically equivalent directions along the two-fold *b*-axis compared to paraelectric α-phase ($TGT\bar{G}$ conformation). Thus, the presence of β-phase in PVDF films is highly desirable as it can provide ferroelectric properties, necessary for sensors, actuators, electro-optical, non-volatile memory and biomedical applications^9^. In addition, ferroelectric poly(vinylidene fluoride-co-trifluoroethylene), P(VDF-TrFE), a copolymer of PVDF, is attractive for device fabrication owing to its more thermodynamically stable β-phase. However, the relatively lower operating temperature range and higher cost of P(VDF-TrFE) restricts its use in large-scale device fabrication^10^. Since, PVDF exhibits a wider operating temperature zone than P(VDF-TrFE)^10^, there have been a number of interesting efforts to induce the nucleation of β-phase in PVDF which includes, electrical poling, mechanical stretching, extreme thermal and pressure conditions annealing and spin coating^11^. However, multistep fabrication techniques often lead to undesired structural deformations or structural limitations and restrict the manipulation of device dimensions and thus the range of applications. Currently, PVDF continues to exhibit relatively low piezoelectric charge co-efficients after such post-processing techniques^8–12^.

An alternative approach to the production of ferroelectric polymers with higher piezoelectric charge co-efficients is to use additives that create a structure that combines a self-poled ferroelectric β-phase and a porous electret structure. This approach has proven useful to achieve superior piezo- and pyro-electric harvesting without the need of a secondary processing methods^1,12^. Among the several additives that have been explored, lanthanide ions such as, Ce^3+^, Eu^3+^, Yb^3+^ etc. have been proven to be useful for facile fabrication of PVDF based high performance piezoelectric nanogenerator (NG)^13–15^. Despite the noticeable optical properties of the lanthanide ions doped PVDF films, they have yet to be used for pyro-electric energy harvesting and sensing. Among the other lanthanide ions, Er^3+^ is known to possess superior optical activity in the infrared (IR) region^16–20^. Infrared rays are known to induce a photo-thermal effect, especially the near infrared rays (NIR), i.e., 760–1500 nm wavelength region^21^. Thus, there is a scope to develop materials for infrared driven pyroelectric energy harvesters for non-contact and remote energy transfer. In addition, thermal sensitivity can be enhanced by integrated design of Er^3+^ and PVDF under an IR irradiation for sensor applications. While thermoelectric materials utilize a spatial temperature difference, pyroelectric energy harvesters and sensors require continuous temperature fluctuations induced by an external heat source in order to develop an alternating electrical current^22^.

Based on these ideas, this paper aims to produce a material with (i) a high fraction of ferroelectric β-phase, (ii) a porous structure to achieve electret-like properties in order to enhance the mechanical energy harvesting ability and (iii) a high optical activity in the IR region for thermal energy harvesting and sensing.

This is achieved by developing a one step approach where a hygroscopic rare earth erbium salt (Er^3+^) is incorporated into a PVDF film (Er-PVDF film). The resulting composite film is shown to contain a thermally stable β-phase even higher stability than a P(VDF-TrFE) film. Owing to its superior ferroelectric and dielectric properties, a high performance ferroelectretic nanogenerator (termed as, FTNG) was fabricated which generated a maximum output power of 0.125 W/m^2^ and 13 µW/m^2^ under mechanical and thermal fluctuations respectively. In addition, FTNG can also act as a healthcare monitoring sensor and thus may be beneficial for further development of self-powered electronic skin (e-skin), sensors and other low power electronic systems.

## Results and Discussion

### Structural and Electrical Properties

**Figure 1:**
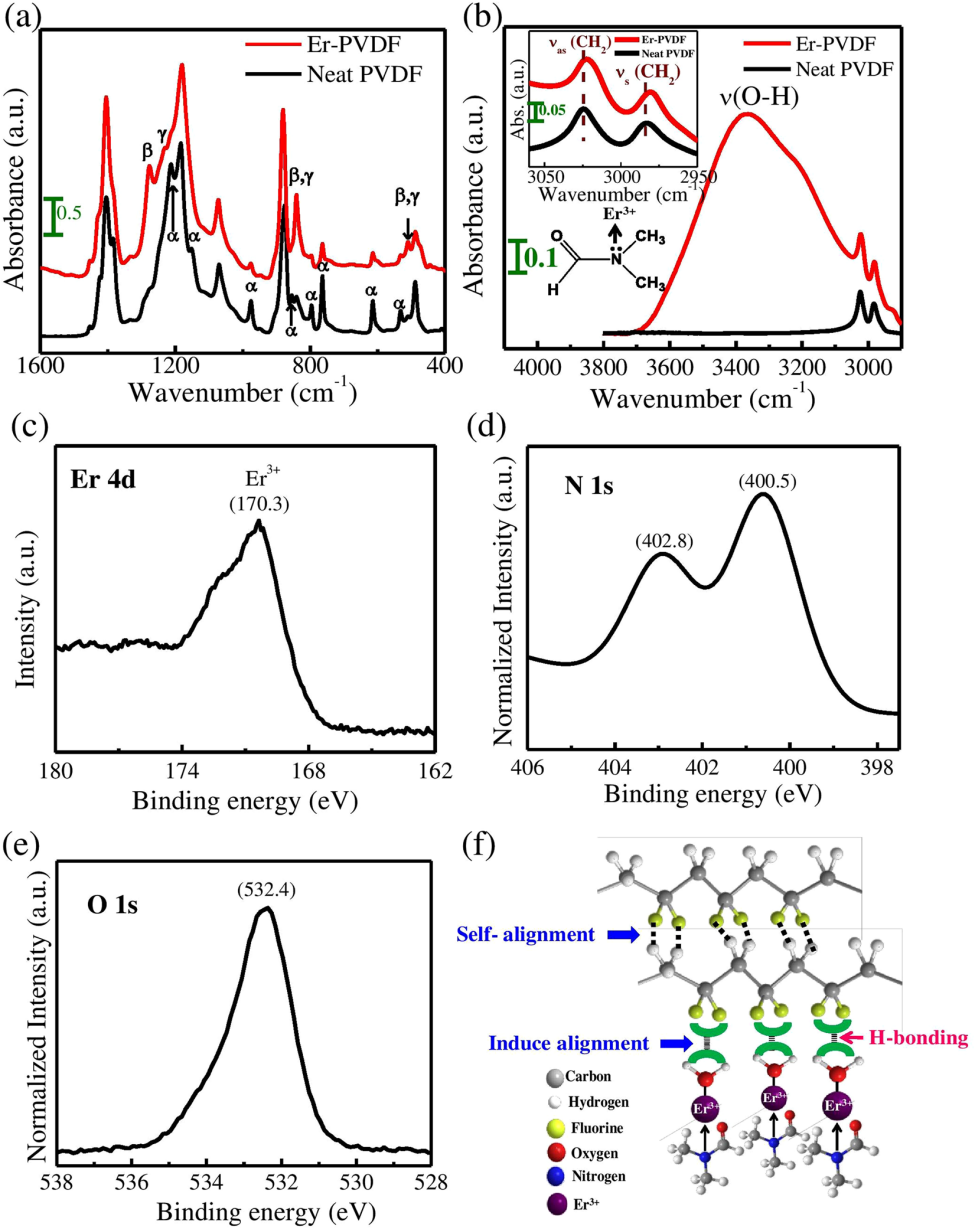
(**a**) FT-IR spectra of the films in the frequency region of (**a**) 1600–400 cm^−1^, (**b**) 3800–2900 cm^−1^ and 3060–2950 cm^−1^ in the inset. The left lower inset in Fig. 1b shows the coordination complex structure of Er^3+^─DMF. High-resolution XP spectra of (**c**) Er 4d, (**d**) N 1 s (**e**) O 1 s and (**f**) schematic illustration of the β-phase nucleation process in the Er-PVDF film.

Figure 1a shows that the Neat PVDF possesses mainly a non-polar α-phase due to the existence of vibrational bands at 1212, 1150, 976, 855, 796, 764, 614 and 532 cm^−1^. However, the Er-PVDF film exhibits four additional peaks at 1276, 1234, 841 and 510 cm^−1^ due to the nucleation of ferroelectric β-and γ-phases^11,23^. Among them, 1276 and 1234 cm^−1^ vibrational bands represent the presence of β-and γ-phases respectively. The peaks at 841 and 510 cm^−1^ are indicative of both ferroelectric phases (*i*.*e*., β-and γ-phases)^23,24^. It is important to identify that the appearance of β-phase (1276 cm^−1^) is strong but the nucleation of γ-phase (1234 cm^−1^) is relatively weak. Quantitatively, the relative proportion of ferroelectric phases (F_EA_) is estimated from Lambert-Beer’s law considering the band at 841 cm^−1^ since it carries the dual signature of the both β-and γ-phases. The F_EA_ is found to be 75% in the Er-PVDF film, estimated using the following equation,

1 $${F}_{EA}=\frac{{I}_{EA}}{(\frac{{K}_{841}}{{K}_{764}}){I}_{764}+{I}_{EA}}\times 100 \% ,$$

where, *I*_764_ and *I*_*EA*_ are the absorbance band intensity at 764 and 841 cm^−1^ respectively; *K*_764_ = 6.1 × 10^4^ *cm* ^2^ *mol* ^−1^ and *K*_841_ = 7.7 × 10^4^ *cm* ^2^ *mol* ^−1^ are the absorption coefficients at the respective wave numbers^11^. An improved β-phase fraction (*F*(*β*) ~ 74%) with a small amount of γ-phase (*F*(*γ*) ~ 1%) in the Er-PVDF film was achieved (Supplementary Fig. S1) when compared to the Neat PVDF which is dominated by the non-polar α-phase^23^.

The mechanism of ferroelectric β-phase nucleation is possibly due to hydrogen bonding interaction viz. O─H-----F─C between the Er-salt and PVDF chains, as a stronger polarity of the hydroxyl groups is evident from the broad absorption band in the frequency region 3800–2950 cm^−1^, see Fig. 1b ^25^. When the Er-salt is mixed with a PVDF-DMF solution, a coordination complex is formed between DMF and Er^3+^, where the N atom is acting as a coordination site of the DMF ligand, as shown in the inset of Fig. 1b. The high-resolution core level spectrum of Er 4d by X-ray Photoelectron Spectroscopy (XPS) shows a distinct peak at 170.3 eV due to the presence of Er^3+^ in the Er-PVDF film as shown in Fig. 1c ^26^. The splitting of the N 1 s spectrum to give peaks around 402.8 and 400.5 eV (Fig. 1d) shows unequivocally, that there are two different N environments in the sample. The peak at 400.5 is consistent with other XPS of DMF (possibly coordinated via its oxygen, or perhaps just trapped in the PVDF)^27^. It is therefore reasonable to suggest that the N (1 s) peak at 402.8 eV is due to N coordinated to the Er, the higher binding energy being consistent with electron donation from the N to the Er. The peak in the O 1 s spectrum at 532.4 eV can be assigned to chemisorbed H_2_O and DMF (Fig. 1e) which is consistent with the strong OH stretch in the FT-IR band between 3800–2950 cm^−1^ (Fig. 1b) ^28,29^. In addition, the stretching vibrational band (ν_C=O_) at 1630 cm^−1^ supports the formation of a Er^3+^–DMF coordination complex (Supplementary Fig. S2) ^28^. Under prolonged stirring of the Er-salt that is incorporated in the PVDF-DMF solution, atmospheric moisture is gradually absorbed due to its polar aprotic and hydrophilic nature. Thus, water molecules from moisture and in co-operation with co-ordination water of the Er-salt (ErCl_3_, 6H_2_O) surround the F^−^ ions of the PVDF. Consequently, the strong hydrogen-bonding interaction of water molecules towards the F^−^ of PVDF (*i*.*e*., O–H-----F–C) drives the –CH_2_/–CF_2_ dipoles so that they are arranged in the all trans (TTTT) configuration (i.e., β-phase), see Fig. 1f. As a consequence, other –CH_2_/–CF_2_ dipoles of PVDF are aligned and the induced alignment within the PVDF by H-bonding interaction further self-aligns the other PVDF macromolecular chains within the crystalline lamella of the Er-PVDF composite film; this process is shown schematically in Fig. 1f. Such an interaction is evident from the shift of –CH_2_ asymmetric (ν_as_) and symmetric (ν_s_) stretching vibrational bands towards a lower frequency, see the inset of Fig. 1b. This is presumably due to an increase in the effective mass of –CH_2_ dipoles during interfacial interaction^23,25^. As a result, damping of the vibrational frequency (*i*.*e*., wavenumber) associated with –CH_2_ stretching occurs that, leads to a damping constant (2*r*_*dc*_) ~4.8 × 10^11^ sec^−1^ which, indicates an improved interfacial interaction to promote an increased β-phase content^23,25^. Eventually, the overall degree of crystallinity (*χ*_*c*_) in the Er-PVDF film reduces (*χ*_*c*_ ~ 38%) in comparison to the Neat PVDF film (*χ*_*c*_ ~ 51%) (Supplementary Fig. S3). The nucleation of β-phase in Er-PVDF is also prominent in the X-ray diffraction pattern, where a sharp diffraction peak at 20.8° can be attributed to the presence of β-phase, and the remaining peaks arise due to presence of α-and γ-phases. In addition, the large crystallite size of the β-crystal (D_β(110)_ ~ 10 nm) (calculated from Debye−Scherrer equation as mentioned in Supplementary Fig. S3) is also beneficial for the molecular dipoles (i.e., –CH_2_ or –CF_2_) to be more cooperative to provide improved ferroelectric properties and dielectric responses^23^.

**Figure 2:**
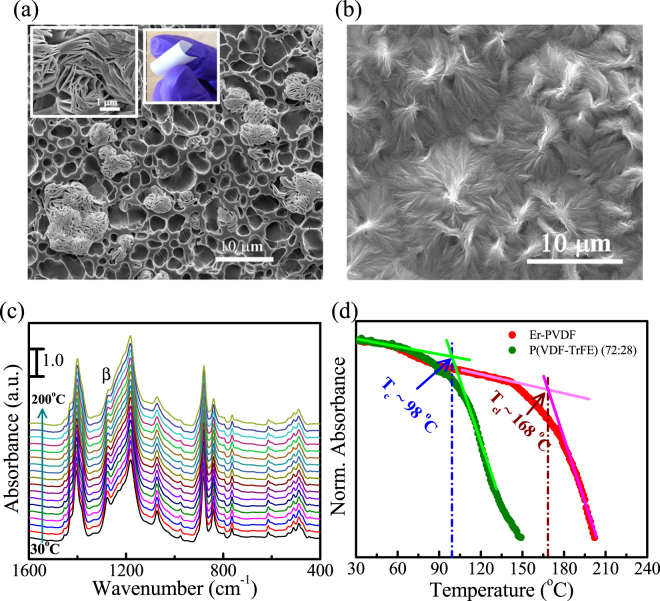
FE-SEM images of (**a**) Er-PVDF film with the enlarged view of the selected portion in the inset as well as digital photographs of the film and (**b**) Neat PVDF film. (**c**) *In-situ* thermal FT-IR spectra of Er-PVDF film, where the spectra between 30 °C to 200 °C are shown in 10 °C intervals to guide the eye. (**d**) The variation of absorbance intensities of the β- phase with respect to the temperature for both Er-PVDF and P(VDF-TrFE) film.

It is important to note that, during crystallization under heat treatment, water molecules evaporate faster than the base solvent (DMF) due to its lower density and lower boiling point. In addition, inorganic salts generally crystallize more rapidly than polymers during the crystallization process due to the high mobility of inorganic ions^30^. Thus, a synergistic effect of the competitive behavior between solvent and non-solvent systems by inhibiting the entanglement among the macromolecular chains of PVDF and fast crystallization of the Er-salt create an electret-like porous structure with a flower-like surface morphology within the Er-PVDF film, see Fig. 2a. The micro-pores are non-continuous and are formed in a layer-by-layer structure up to certain depth (~5 µm); see Supplementary Fig. S4. In contrast, the Neat PVDF film exhibits a smooth surface with several fibril-like α-spherulitic growths as seen in Fig. 2b. The Er-PVDF film appears to be highly flexible due to the porous structure, see inset of Fig. 2a. In addition, the porous structure is also beneficial for superior ferroelectricity and piezoelectric output performance^12,15,31,32^.

In order to examine the thermal stability of ferroelectric β-phase in Er-PVDF (Fig. 2c and Supplementary Fig. S5), the absorption band at 1276 cm^−1^ has been considered as it is indicative of only the β-phase^23–25^. For comparison a P(VDF-TrFE) film is selected since it has a well-defined Curie transition temperature (T_c_) (Supplementary Fig. S6). Figure 2d shows the relative change of absorbance of vibrational band as a function of temperature. It has been observed that β-phase in Er-PVDF film exhibits higher thermal stability than that of the P(VDF-TrFE) film. For instance, the ferroelectric to paraelectric phase transition occurs at 98 °C in the P(VDF-TrFE) film which is the T_c_^10^. In contrast, a similar phase transition (T_cl_) is observed at a much higher temperature of 168 °C in the Er-PVDF film. Furthermore, in the vicinity of the T_c_, the rate of change of absorbance as a function of temperature in Er-PVDF film is less steeper than the P(VDF-TrFE). These results indicate that the operating temperature region is expected to be higher in devices made with Er-PVDF composite since at the vicinity of T_c_ the chain mobility of PVDF is significantly enhanced that eventually leads to a ferroelectric to paraelectric transition^10,33^. Since the Er-PVDF film is mainly composed of the all-trans β-phase, which is thermodynamically stabilized with interfacial interaction phenomena and H-bonding, the T_c_-like (T_cl_) phase transition temperature is significantly improved (~62%). This provides a route to fabricate more reliable and thermally robust PVDF based electronic devices.

**Figure 3:**
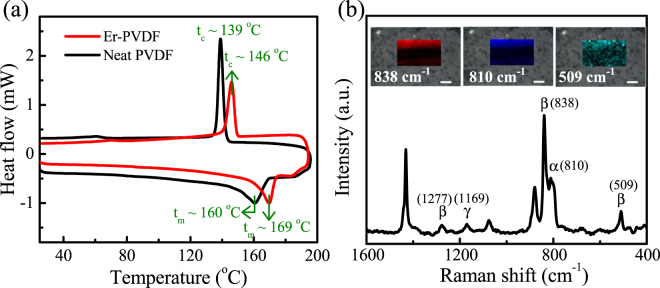
(**a**) DSC thermograms of the Er-PVDF and Neat PVDF films, (**b**) Raman spectra of Er-PVDF film with the mapping images at 838 (red mapping), 810 (blue mapping) and 509 cm^−1^ (dark cyan mapping).

In order to further understand the effect of Er^3+^ on the thermal stabilization and crystallization of PVDF, differential scanning calorimetry (DSC) has been performed, see Fig. 3a. It is evident that Er^3+^ increases the melting temperature (t_m_) of the Er-PVDF film (t_m_ ~ 169 °C), compared to the Neat PVDF film (t_m_ ~ 160 °C). It can also be seen that the Er-PVDF film exhibits a higher crystallization temperature (t_c_ ~ 146 °C) compared to the Neat PVDF film (t_c_ ~ 139 °C). This is due to the fact that Er^3+^–DMF coordination complex is acting as a nucleating agent that restricts the movement of PVDF chain segments that lead to an increase in the t_c_ value. In addition, the ${\chi }_{{c}_{t}}$ of the films was further confirmed from the DSC data using, ${\chi }_{{c}_{t}}=\frac{{\rm{\Delta }}{H}_{m}}{(1-\phi ){\rm{\Delta }}{H}_{m}^{0}}$ where, Δ*H*_*m*_ is the melting enthalpy, ${\rm{\Delta }}{H}_{m}^{0}$ is the melting enthalpy of the 100% crystalline PVDF (~104.5 Jg^−1^) and φ is the weight fraction of Er-salt^34^. This calculation revealed ${\chi }_{{c}_{t}}$ ~ 37% for Er-PVDF and ${\chi }_{{c}_{t}}$ ~ 50% for Neat PVDF which are in good agreement with the XRD analysis. In order to map the conformational and structural changes over the large area of Er-PVDF film, we measured the Raman spectra at each point on a 22 × 13 grid spanning an area of 38 µm × 23 µm, see Fig. 3b. The intensity of the Raman bands at 838 and 509 cm^−1^ representing β-phase and at 810 cm^−1^ demonstrating α-phase mapped over the area of 38 µm × 23 µm which correspond to red, blue, and dark cyan images, respectively, in the inset of Fig. 3b ^35,36^. In addition, a very weak band at 1169 cm^−1^ corresponding to the γ-phase was also observed which agrees with the observation made from FT-IR spectra (Fig. 1a)^36^. In contrast, the Neat PVDF film shows only α-phase in Raman spectra (Supplementary Fig. S7). Thus, the Raman spectra were found to be consistent with the FT-IR spectra. Furthermore, the representative mapping images of the Raman spectra indicate that β-crystal domains were mixed with the α-crystal domain in the Er-PVDF film.

**Figure 4:**
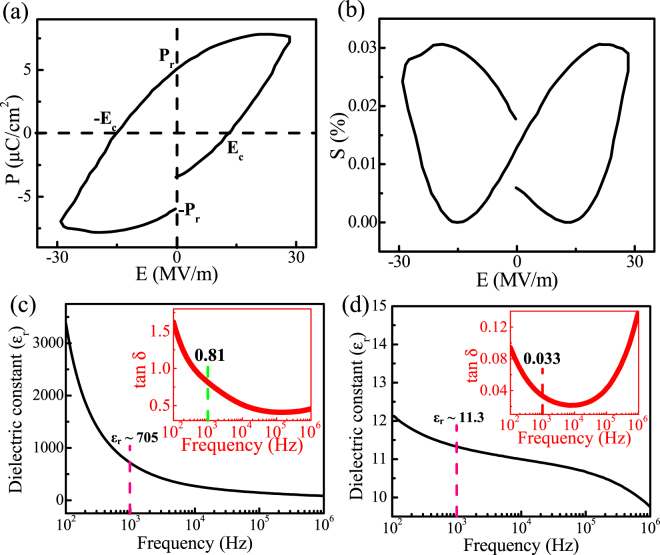
(**a**) Polarization (P) versus electric field (E), (**b**) butterfly shaped strain (S) versus electric field (E) hysteresis loops, (**c**) frequency dependent dielectric spectra of the Er-PVDF film and (**d**) the Neat PVDF film.

As piezoelectricity stems from the level of polarization of a material, the polarization behavior in the Er-PVDF film is examined from the polarization (P) versus electric field (E) hysteresis loop at a frequency of 10 Hz under a sweeping electric field (E) of ± 30 MVm^−1^; as shown in Fig. 4a. The data demonstrates that the porous Er-PVDF film possesses superior remnant polarization, P_r_ ~ 5.12 µCcm^−2^ with a coercive field (E_c_) of 14.4 MVm^−1^; this is in contrast to the Neat PVDF film which exhibits little ferroelectric switching (Supplementary Fig. S8). In addition, the electric field (E) induced strain amplitude (S_a_) response arises from the converse piezoelectric effect and is demonstrated by the presence of butterfly shaped S_a_-E hysteresis loops; see Fig. 4b ^37,38^. According to the macroscopic dimensional effect, the piezoelectric charge coefficient (d_33_) can be determined as, *d*_33_ = *P*_*r*_ *S*_33_ = −56.3pC/N where *S*_33_ (~1.1 × 10^−9^ m^2^N^−1^) is the elastic compliance^15,31^. In addition, from the S_a_-E loop, the longitudinal electrostrictive coefficient (Q) can be evaluated as, S_a_ = QP^2^ = 5 m^4^C^−2^ from the slope of S_a_ versus P^2^ plot (Supplementary Fig. S9). The P-E and S_a_-E hysteresis loops indicate the preferential alignment of electric dipoles within the crystalline lamellas along the direction of the applied electric field. In addition, this may be attributed to the coupling effect of ferroelectric β-phase as nano-dimensional dipoles (nano-dipole) and ferroelectret properties of micro-pores behaving as micro-dipoles under the applied electric field^15,31^. In general, to generate electrets, charges are artificially injected into macroscopic voids of porous polymers to create oriented, “quasi-dipoles”. In this case, a large amount of charge is trapped within the porous structure at dielectric boundaries, such as adjoining crystalline/amorphous regions interfacial polarization, even without any electric poling treatment. The electrons are present through delocalization in the Er^3+^ just like space charge, which removes the need for extra charge injection. In addition, the Er^3+^ influences charge mobilization in the Er-PVDF, and thereby facilitate hetero-polarization in the material. Therefore, during fabrication, charge separation has occurred and oriented -CH_2_/-CF_2_ dipoles stabilize the separated trap charges due to the self-induction effect. This functionality combines the ferroelectric and ferroelectret properties which makes the Er-PVDF film a ‘ferroelectretic’ material^12,15,31^.

In addition, this co-operative functionality also improves the dielectric properties of the Er-PVDF film compared to the Neat PVDF film. For example, a higher dielectric constant, *ε*_*r*_ ~ 705 at 1 kHz has been observed in the Er-PVDF film (Fig. 4c) than that of the Neat PVDF film (*ε*_*r*_ ~ 11.3) (Fig. 4d). However, the loss tangent in Er-PVDF film (tan δ ~ 0.81 at 1 kHz) (inset of Fig. 4c) was increased compared to the Neat PVDF film (tan δ ~ 0.033 at 1 kHz) (inset of Fig. 4d), indicating an increase in conductivity. The dielectric spectra of the Er-PVDF film show that among the degree of polarizations, interfacial and ionic polarization mainly contributes for the increment of dielectric constant. The frequency dependent high dielectric constant (*ε*_*r*_) can be an indicator of some conductivity of the material, which may also explain the higher tan δ in the Er-PVDF film^39^. The possible effects for the increment of *ε*_*r*_ are as follows, (i) presence of micropores acting like microcapacitors^31^, (ii) reduction in the degree of crystallinity (χ_c_) (resulting 33% of the β-crystals) according to Fröhlich’s amorphous breakdown theory^40^, (iii) presence of H-bonding interaction^41^ and (iv) the influence of Er^3+^ ions in the Er-PVDF film which serve as a ionic charge carriers with low mobility^42^. The movement of charge along with the applied electric field leads to charge separation within the electrically neutral dielectric and developing strong electrode effects at the external dielectric-metal interface or Maxwell-Wagner polarization at internal interfaces. Thus, higher dielectric properties along with excellent piezoelectric and electrostrictive co-efficients make the Er-PVDF film an excellent ferroelectretic material for mechanical and thermal energy harvesting applications.

### Piezoelectric Energy Harvesting

**Figure 5:**
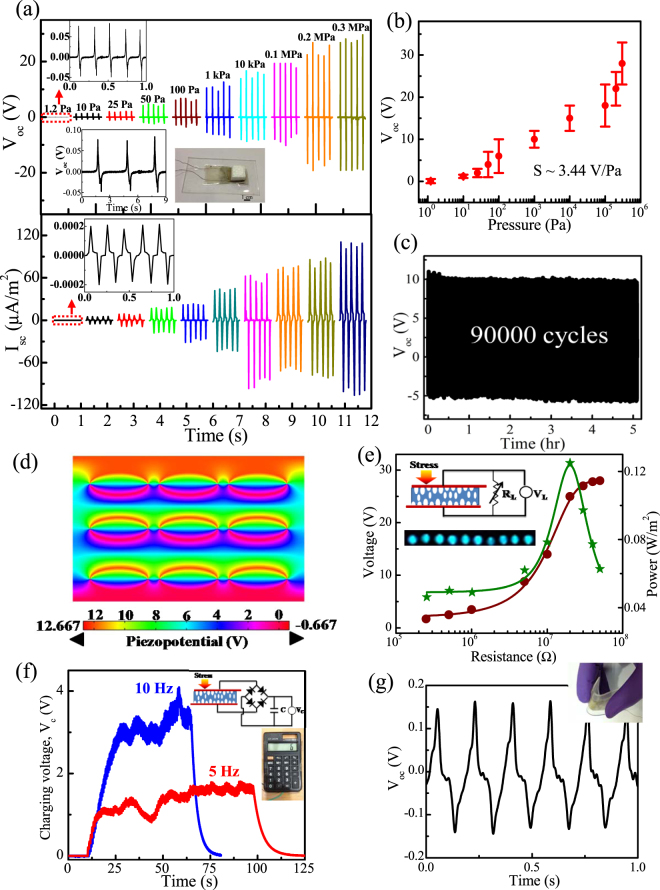
(**a**) Time-dependent open-circuit output voltage (V_oc_), short-circuit output current (I_sc_) under different pressure, (**b**) variation of V_oc_ as a function of pressures, (**c**) energy harvesting stability test over 90000 cycles under 1 kPa pressure at 5 Hz frequency, (**d**) simulation on piezo-potential distribution with deformed nine micro-voids nestled within PVDF under 1 kPa pressure, (**e**) output voltage and power as a function of external load resistances using the electronic circuit diagram and illuminated 10 LEDs in the insets, (**f**) transient response of 1 µF capacitor during voltage charging-discharging cycles under the oscillation frequencies of 5 and 10 Hz using the equivalent circuit diagram shown in the inset and operated digital calculator during discharging of the capacitor shown in the inset and (**g**) generated V_oc_ during repeated bending-unbending of FTNG used for sensing purpose.

Owing to the superior piezo-electret effect, the open circuit output voltage (V_oc_) and short-circuit output current (I_sc_) from FTNG was evaluated by imparting a periodic compressive stress (σ_a_) using a custom built pressure imparting system, as discussed earlier^43^. As shown in Fig. 5a, the produced V_oc_ from FTNG ranges from 70 mV to 28 V when subjected to a stress from σ_a_ ~ 1.2 Pa to 0.3 MPa and a corresponding strain rate of 6.6 × 10^−7^ to 0.165% s^−1^ (Supporting Information, Table S1). In addition, the generated I_sc_ ranges from 0.03 µA/m^2^ to 105 µA/m^2^ under the same applied pressure and strain rate range. An almost linear increase of V_oc_ with increasing σ_a_, see Fig. 5b, provides a quantitative value of sensitivity (S_M_) as, ${S}_{M}=\frac{{\rm{\Delta }}{V}_{oc}}{{\rm{\Delta }}{\sigma }_{a}}=3.4\,{\rm{V}}/{\rm{Pa}}$ where, ΔV_oc_ and Δσ_a_ are the differences of V_oc_ and σ_a_ respectively^43^. In addition, the change of I_sc_ can be attributed to the variation of applied strain rate, because they are directly related to each other as, ${{\rm{I}}}_{{\rm{sc}}}={{\rm{d}}}_{33}\mathrm{YA}\dot{{\rm{\varepsilon }}}$ where Y is the Young’s modulus, A is the effective contact area and $\dot{{\rm{\varepsilon }}}$ is the strain rate^31,38^. Thus, the FTNG is proven to be a highly sensitive energy harvester and was able to detect small dynamic pressure during repeated loading–unloading of a light weight object (such as, a cube of polystyrene foam of 50 mg); as shown in the inset of Fig. 5a. The output voltage and current waveforms show synchronous alteration of positive–negative pulses with repeated compress–release of FTNG under periodic mechanical impacts. In addition, the FTNG shows reverse polarity of V_oc_ when switching the electrode connections (Supplementary Fig. S10) to undertake a ‘switching polarity test’ in order to confirm that the output responses arise due to the piezoelectric effect, and not from any contact electrification between the measurement set-up and device^43^. Furthermore, when two FTNGs of the same polarity and similar electromechanical responses were connected in serial and parallel configurations, the observed V_oc_ ~ 51 V increased in the first case and remained almost same V_oc_ ~ 30 V in second case, respectively (Supplementary Fig. S10). This further demonstrates the piezoelectric effect and also the integratability feature of the FTNG by linear superposition tests where we can modulate the output power. Additionally, as a proof of confirmation a non-piezoelectric PDMS control device (without an Er-PVDF film) was also fabricated where no reliable output voltage is obtained (Supplementary Fig. S11). The superior energy harvesting performance of the FTNG over the previously reported piezoelectric polymer based NGs^5,6^ is attributed to the superior piezoelectric figure of merit (*FoM*_*p*_ ≈ *d*_33_.*g*_33_ ≈ 5.08×10^−13^ *Pa* ^−1^ where, ${g}_{33}=\frac{{d}_{33}}{{\varepsilon }_{r}{\varepsilon }_{0}}=9\times {10}^{-3}\,{{\rm{VmN}}}^{-1}$) and intrinsic piezoelectricity of the ferroelectret characteristics due to the spongy nature of micro-voids in comparison to the stiffer surrounding PVDF matrix^15,31,44^. For ferroelectric PVDF, the local polarization is compensated by space charges around the crystallites and the interconnected amorphous regions behave like a spring. Thus, under an external mechanical impact (σ_a_), the deformation of the micro-voids in comparison to the PVDF chain is considerably larger due to the relatively weak vander Waal bonds as well as electrostatic interactions between the macromolecular chains in comparison to the strong covalent bonds within the chain which induces piezoelectric potential within FTNG^31^. As a result, free electrons move to the external load in order to screen the piezo-potential which leads to a positive voltage peak. On release of the applied stress, σ_a_, negative voltage distributions are generated due to a decrease of the piezo-potential and release of the accumulated electrons. Importantly, the FTNG demonstrates a highly stable generation of V_oc_ over prolonged period of time (90000 cycles) under 1 kPa pressure at 5 Hz, see Fig. 5c, due to robust mechanical property of the nanocomposite structures under significant deformation and the flexibility of the entire structure. The contribution of the highly deformed micro-voids to the piezo-voltage generation of the FTNG was quantitatively explained by the finite element method (FEM) based simulation using COMSOL multiphysics software with nine micro-voids placed into a PVDF structure (see Fig. 5d). The piezo-potential distribution inside the deformed FTNG is represented by the color contour with a z-axis mechanical stress (σ_a_ ~ 1 kPa) and the magnitude of the simulated piezopotential (~12 V) is almost consistent with the experimental finding (~10 V). The effective piezoelectric power output from FTNG was calculated by measuring the output voltages across various load resistors (R_L_) ranging from 0.25 MΩ to 50 MΩ, as seen in Fig. 5e, using a simple electronic circuit (inset of Fig. 5e). The measured instantaneous voltage drop (V_L_) across the resistors gradually increases with an increase of R_L_ and saturates at very high resistances (~50 MΩ) corresponding to the open circuit voltage. The effective output power density (P) of the FTNG is calculated by ${\rm{P}}=\,\frac{1}{{\rm{A}}}.\frac{{{{\rm{V}}}_{{\rm{L}}}}^{2}}{{{\rm{R}}}_{{\rm{L}}}}$ where, A is the effective contact area and V_L_ is the voltage drop across the load resistance R_L_. Thus, an estimated instantaneous output power density is 0.125 W/m^2^ at an R_L_ of 20 MΩ which is shown to be sufficient to turn on several (~10) blue LEDs when subject to a mechanical touch without any storage system; see inset of Fig. 5e. In order to provide more a useful application of the FTNG, the device was connected to an external capacitor through a full-wave bridge rectifier, as shown in Fig. 5f. It is interesting to note that the FTNG was able to charge up a capacitor of 1 µF capacitance up to 4 V within 70 s when impacted by 10 Hz frequency mechanical load and a higher frequency yielded faster charging of the capacitor. For example, a lower voltage (~1.8 V) was reached across 1 µF capacitor under 5 Hz frequency impact. During discharging, the stored maximum energy (E = $\frac{1}{2}C{V}_{c}^{2}$ =  8µJ) across the capacitor was further used to drive a digital calculator, see the inset of Fig. 5f. Owing to the high sensitivity of the device, bending and releasing of the FTNG in a cyclical manner using a human finger also generates V_oc_ ~ 150 mV (Fig. 5g). In this case, a tensile strain is developed along the thickness direction (${\varepsilon }_{y}$, parallel to the dipole orientation) during bending of the device into an arc shape which is given by, ${\varepsilon }_{y}=\frac{L}{2r}=0.433\, \%$, where, *r* = 15 *mm* is the bending radius and L is the thickness (~130 μm)^15,31^. Alternatively, the strain developed along the length direction (perpendicular to the preferential dipole orientation) is ${\varepsilon }_{x} \sim$ 0.190%, obtained from the relation of Poisson’s ratio, $\nu =|\frac{{\varepsilon }_{x}}{{\varepsilon }_{y}}|=\,0.44$ of PVDF^15^. This result includes the static tactile sensing ability of FTNG which can be helpful for physiological signal monitoring.

### Wearable Healthcare Monitoring System

**Figure 6:**
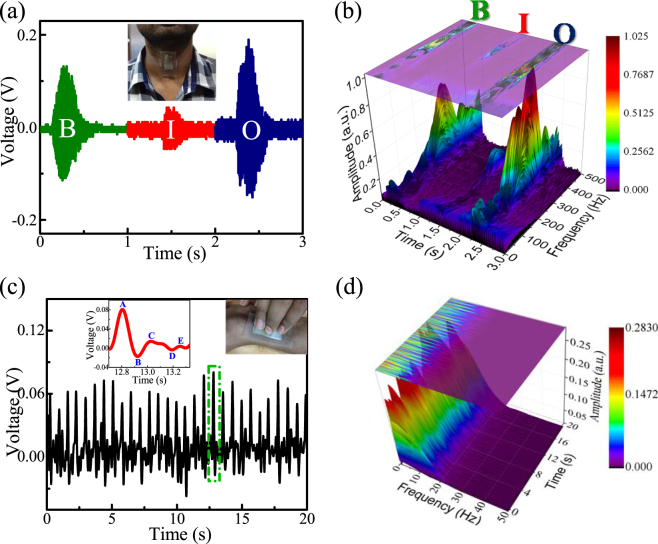
(**a**) Generated output voltage signals during speaking of the alphabets, “B”, “I” and “O” after attaching the FTNG to the throat and (**b**) STFT processed 3D spectrogram of the speech patterns, recognizing the frequency range of each word. (**c**) Wrist pulse signal detection by fastening the FTNG onto the human wrist directly (enlarge view of one signal showing details of wrist pulse) and (**d**) corresponding STFT processed 3D spectrogram for further analysis of the heartbeat signals.

As a wearable sensor, the FTNG was attached to a human throat by adhesive tape as shown in Fig. 6a. The device could recognize the vibration of vocal cords and generate a corresponding time dependent output voltage waveform in response to the pronunciation of various letters of the alphabets such as “B”, “I”, and “O” from a speaker. An alternative way to view the data is represented by short-time Fourier transform (STFT) processed 3D spectrograms, in which the spectral content appears in a color contour plot with time, frequency and amplitude along the x, y and z axes, respectively (Fig. 6b). The acoustic profile of the alphabets shows that maximum amplitude of “B” occurs around 350 Hz, “I” appears around 375 Hz and “O” lies around 400 Hz. Thus, FTNG could be non-invasively used to monitor damaged vocal cord and in speech rehabilitation training^43^.

In addition, when the FTNG was firmly attached to a human wrist, as shown in Fig. 6c, it could successively sense the subtle pressure changes of the radial artery blood pressure. At the rest condition, the FTNG can read out a real time heart beat (~84 beats per minute) where each peak denotes one pulse. Details of the heart beat can be found by analyzing each voltage output waveforms which is composed of mainly five parts, such as, initially positive (A-wave), early negative (B-wave), reincreasing (C-wave), late redecreasing (D-wave), and diastolic positive (E-wave)^45^. The important clinical insights from heart beat signals could be assessed from the corresponding STFT processed spectrogram, see Fig. 6d. These results show that each heart beat pulse lies within the frequency range of 0–20 Hz and this frequency domain features could be further used in order to determine the age related heart condition^43,45^. Thus, the potential of FTNG as a self-powered healthcare monitoring electronic skin (e-skin) could pave the way for next generation self-powered wearable biomedical electronic systems^46^.

### Pyroelectric Energy Harvesting

**Figure 7:**
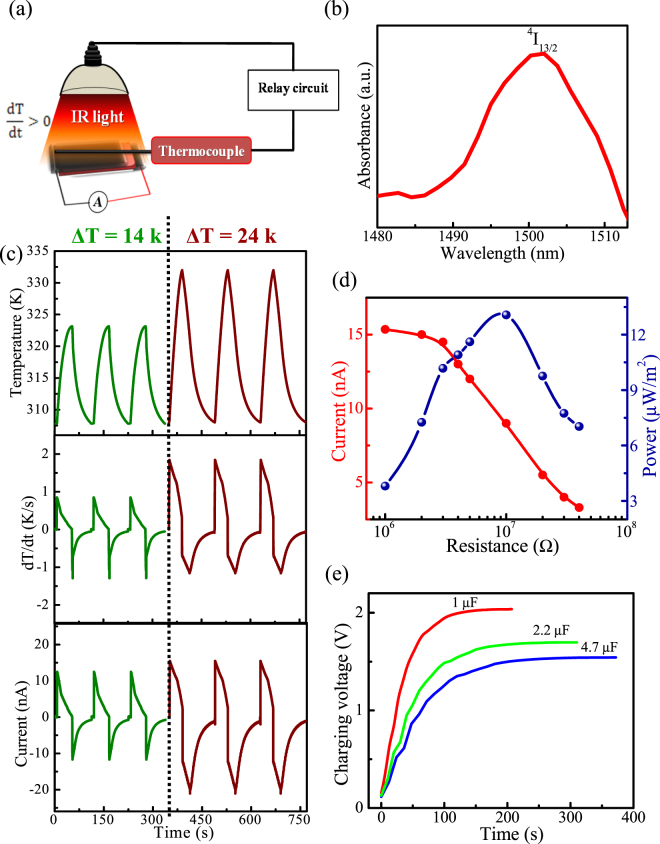
Pyroelectric output responses of FTNG. (**a**) Schematic of pyroelectric experimental setup. (**b**) Absorbance spectra of Er-PVDF film in the 1480–1513 nm wavelength range. (**c**) The time-dependent cyclic changes in the different range of applied temperatures and temperature gradient (dT/dt) which generate short-circuit currents. (**d**) Output short-circuit current and power density as a function of external resistances varying from 1 MΩ to 40 MΩ. (**e**) Transient responses of external capacitors (1 µF, 2.2 µF and 4.7 µF) charging under heating-cooling cycles of the temperature difference ΔT ~ 24 K.

The ferroelectretic property of the Er-PVDF film also enables the FTNG to harvest thermal energy by using the pyroelectric effect. Temperature oscillations were applied to the FTNG by illumination of IR light to assess the pyroelectric short circuit current (I_psc_). The design for the pyroelectric energy harvesting system is shown schematically in Fig. 7a. Interestingly, the Er^3+^ containing Er-PVDF film is a good absorber of IR light, as shown by the absorption spectra in Fig. 7b. Thus, the material is attractive for direct thermal to electrical energy conversion which enables small-scale energy harvesting devices to operate at low temperatures. The absorption peak around 1502 nm corresponds to the transition from the ground state ^4^I_15/2_ to excited state ^4^I_13/2_^16,18–20^. Thus, the FTNG was placed below an IR light bulb at a 2 cm distance. The developed temperature gradient was measured using a thermocouple at the surface of the FTNG by contact conduction. When the light was switched on to heat the FTNG, the temperature increased from 307 K to a higher value and an increase of the generated I_psc_ was observed. When the light was switched off, the device cooled down and a decrease of I_psc_ was observed. Figure 6c shows that under the exposure of temperature changes from ΔT ~ 14 K to 24 K, an increase of I_pcs_ ~ 12.5 nA to 15.5 nA have been observed. This enhancement was achieved by a thermally induced piezoelectric coupling effect, as the output performance is proportional to Δ*T* and dT/dt^47^. Under a mechanical stress (σ) and strain (S), pyroelectrics also experience a change in spontaneous polarization ($d{P}_{{s}_{ij}}$) as they are a subgroup of piezoelectric materials. The application of heat results in an applied stress and eventually, thermal strain in the Er-PVDF film. Thus, the $d{P}_{{s}_{ij}}$ is a combination of its primary pyroelectric coefficient *p* and secondary contribution (${d}_{ij}{c}_{ijk}{\alpha }_{i}$) due to thermal strain and piezoelectric contribution. Therefore, in case of metal-insulator-metal (MIM) structure, the change in polarization, $d{P}_{{s}_{ij}}$ with respect to the change in temperature dT can be written as,

$$\frac{d{P}_{{s}_{ij}}}{dT}=p-{d}_{ij}{c}_{ijk}({\alpha }_{i}-\frac{d{S}_{ij}}{dT})$$

where, *p* is the pyroelectric co-efficient (Cm^−2^K^−1^), d_ij_ (C/N) is the piezoelectric coefficient, c_ijk_, (N/m) is mechanical stiffness, α_i_ is material thermal expansion, and dS_ij_/dT is thermally induced strain^48^.

In addition, the IR ray absorbing capability of the Er-PVDF film enhances heat transfer and achieves both faster and larger temperature fluctuations, which improves pyroelectric energy transformations. The origin of the pyroelectric effect in the FTNG stems from the ferroelectretic properties of Er-PVDF film because porosity significantly decrease the specific heat capacity and increases the pyroelectric properties for energy harvesting^44^. The pyroelectric figure of merit can be expressed as, $Fo{M}_{py}=\frac{{p}^{2}}{\varepsilon {C}_{E}^{2}}$ where, $\varepsilon ={\varepsilon }_{0}{\varepsilon }_{r}$ is the permittivity and ${C}_{E}$ is the volume specific heat and derived by ${C}_{E}=\rho {C}_{p}$, where ${C}_{p}$ is the measured specific heat capacity and $\rho$ ~ 1.74 g.cm^−3^ (according to data sheet)^44^. The Er-PVDF film possesses lower specific heat capacity than the Neat PVDF (Supplementary Fig. S12) which implies its higher thermal energy harvesting capability. Thus, Er-PVDF film exhibits a $Fo{M}_{py}=0.05\,\,$pm^3^J^−1^. In fact, the level of polarization (P) changes as a result of a change in temperature dT (K). When the level of polarization decreases on heating, surface bound charge become free and this creates an electric field across the polar axis^49^. This potential difference can be discharged across an external load when the surface electrodes of the FTNG are interconnected via external load. Under short circuit conditions, the pyroelectric current is given by ${{\rm{I}}}_{{\rm{psc}}}={\rm{A}}\frac{{\rm{dP}}}{{\rm{dT}}}\frac{{\rm{dT}}}{{\rm{dt}}}={\rm{A}}p\frac{{\rm{dT}}}{{\rm{dt}}}$ where, A is surface area (m^2^) and $\frac{{\rm{dT}}}{{\rm{dt}}}$ is the rate of temperature change (Ks^−1^)^1,44,47–50^. Thus, the pyroelectric co-efficient of the FTNG is 33 µCm^−2^K^−1^. The pyroelectric output currents (I_pL_) were further measured under ΔT ~ 24 K across variable external resistances (R_L_) ranging from 1 MΩ to 40 MΩ (Fig. 7d). The output power (P) was evaluated using the relation, ${\rm{P}}={{\rm{I}}}_{{\rm{pL}}}^{2}\times {{\rm{R}}}_{{\rm{L}}}$. The maximum pyroelectric output power was found to be 13 µWm^−2^ across the resistance of 10 MΩ. In order to demonstrate the practical application of the FTNG as a pyroelectric energy harvester, the device was used to charge up commercial capacitors of a range of capacitances such as, 1 µF, 2.2 µF and 4.7 µF (Fig. 7e). Under repeated heating–cooling cycles, the FTNG stored 1.6 µJ, 1.1 µJ and 2.3 µJ of energy ($E=\frac{1}{2}C{V}_{c}^{2}$ where C is the capacitance and V_c_ is the charging voltage) across 1 µF, 2.2 µF and 4.7 µF capacitors respectively. The stored energy could be further used to drive the low power consumer electronics. This indicates that FTNG could be applicable to harvest waste heat energy as well as mechanical vibrational energy to operate our low power consumer electronic devices in addition to smart biomedical devices for self-powered wearable electronics and e-skin applications.

## Conclusion

We have demonstrated a novel infrared sensitive Er^3+^ modified poly(vinylidene fluoride) (PVDF) (Er-PVDF) film whose piezo- and pyro-electric properties are designed for superior harvesting of both mechanical and thermal energies. The Er^3+^ is shown to provide enhanced nucleation of ferroelectric β-phase and aids in the formation of an electret-like porous structure to provide the improved piezoelectric properties. In addition, due to its excellent sensitivity to infrared light, the Er^3+^ enhances heat transfer in the Er-PVDF film and provides rapid and large temperature fluctuations during heating, thereby improving pyroelectric energy transformation. As a new form of hybrid harvesting material and mechanical and thermal sensor, it is shown that the combination of the rare-earth Er^3+^ ion with the ferroelectricity of PVDF can find a broad range of applications in self-powered personal microelectronics, temperature and motion sensing, and *e*-healthcare monitoring.

## Methods

### Film preparation

In our experiment, we added 2.0 wt % (w/v) of erbium (III) chloride hexahydrate (ErCl_3_, 6H_2_O, Sigma–Aldrich, USA) salt (i.e., Er-salt) to a 6 wt % (w/v) of PVDF (Sigma–Aldrich, USA)–N,N-dimethyl formamide (DMF, Merck, India) solution and stirred at room temperature. The film was deposited by a solution casting process at 120 °C for 8 h under vacuum conditions. As a reference, pure PVDF (termed as, Neat PVDF) and P(VDF-TrFF) (72:28 mol%) films were also prepared under similar conditions. The films were named as Er-PVDF (where Er-salt is present) and Neat PVDF (where no additive is present).

### Nanogenerator fabrication

In order to fabricate the FTNG, top and bottom electrodes (effective area, A: 250 mm^2^) were made with silver paste. Electrical output leads were attached to the each side of the electrodes by means of silver paste. Finally, the electrode-film sandwich structure was encapsulated with a poly(dimethylsiloxane) (PDMS) (Sylgard 184 silicone elastomer) layer (~10 µm thickness) to increase the robustness of the device. The PDMS was prepared by mixing the silicone elastomer with curing agents in the ratio of 10:1 and heated at 60 °C for 30 min after degassing.

### Material characterization

The crystalline modifications and *in-situ* thermal stability (thermal ramp 1 °C/min) of the films were analyzed by Fourier Transform Infrared Spectroscopy (FT-IR, Tensor II, Bruker). Morphological features were investigated by Field Emission Scanning Electron Microscopy (FE-SEM, FEI, INSPECT F 50). The degree of crystallinity in the Er-PVDF and Neat PVDF films were estimated by X-ray diffraction analysis (XRD, D8 Advance, Bruker) with CuKα, X-ray radiation (λ = 0.154178 nm). Differential scanning calorimetry (DSC) was performed (microSC, Setaram) from 20 to 200 °C at a heating rate of 1 °C.min^−1^. The specific heat capacity (C_p_) of the films was measured from 20 to 60°C by a MicroSC multicell calorimeter from Setaram, with the Calisto program to collect and process the data. Raman spectra were recorded using a Raman microscope (Renishaw inVia) using 785 nm excitation from a HeNe laser in the range 1600–400 cm^−1^. The sample was excited by 7 mW incident laser power, and scattered light was collected through a 50 × objective lens (Leica, numerical aperture (NA) = 0.75) with 2 μm spot size. We measured the Raman spectra at each point on 22 × 13 grids over an area of 38 µm × 23 μm and mapped the intensity of Raman bands at 810, 838 and 509 cm^−1^ over the entire region. Each data point resulted from an exposure time of 25 s. A Kratos Axis Ultra DLD system was used to collect XPS spectra using monochromatic Al *K*_*α*_ X-ray source operating at 150 W (10 mA × 15 kV). Data was collected with pass energies of 80 eV for survey spectra, and 40 eV for the high resolution scans with step sizes of 1 eV and 0.1 eV respectively. The system was operated in the Hybrid mode, using a combination of magnetic immersion and electrostatic lenses and acquired over an area approximately 300 × 700 µm^2^. A magnetically confined charge compensation system was used to minimize charging of the sample surface, and all spectra were taken with a 90° take of angle. A base pressure of ~1 × 10^−9^ Torr was maintained during collection of the spectra. Data was analysed using CasaXPS (v2.3.19rev1.1 l) after subtraction of a Shirley background and using modified Wagner sensitivity factors as supplied by the manufacturer. The optical property of the Er-PVDF film has been studied by NIR spectrometer (DWARF STAR, StellarNet Inc.) within 900–1700 nm wavelength range. The polarization (P) versus electric field (E) hysteresis loops were measured from the ferroelectric testing system (P-E, P-LC100V, Radiant Technology Precision) connected to a high voltage interface, employing a bipolar triangular electric field (E). Frequency dependent dielectric properties were measured from a 20 Hz to 10 MHz frequency range using a precision impedance analyzer (Wayne Kerr, 6500B). The output voltages and currents of the FTNG were recorded using digital storage oscilloscope (Tektronix, TDS2024C) and picoammeter (Keithley 6485) respectively. The vertical forces were recorded by a 3-axial force pressure sensor (FlexiForce A201).

### Data Availability

The datasets generated during and/or analysed during the current study are available from the corresponding author on reasonable request.

## Electronic supplementary material

Supplementary information

## References

[1] Bowen CR, Kim HA, Weaver PM, Dunn S (2014). Piezoelectric and ferroelectric materials and structures for energy harvesting applications. Energy Environ. Sci..

[2] Zhang J, Wang C, Bowen C (2014). Piezoelectric effects and electromechanical theories at the nanoscale. Nanoscale.

[3] Yoon S, Sim JK, Cho YH (2016). A flexible and wearable human stress monitoring patch. Sci. Rep..

[4] Alam MM, Ghosh SK, Sultana A, Mandal D (2015). Lead-free ZnSnO3/MWCNTs-based self-poled flexible hybrid nanogenerator for piezoelectric power generation. Nanotechnology.

[5] Hou C (2013). A strong and stretchable self-healing film with self-activated pressure sensitivity for potential artificial skin applications. Sci. Rep..

[6] Chen X (2015). A high performance P(VDF-TrFE) nanogenerator with self-connected and vertically integrated fibers by patterned EHD pulling. Nanoscale.

[7] Saito Y (2004). Lead-free piezoceramics. Nature.

[8] Wan C, Bowen CR (2017). Multiscale-structuring of polyvinylidene fluoride for energy harvesting: the impact of molecular-, micro- and macro-structure. J. Mater. Chem. A.

[9] Lovinger AJ (1983). Ferroelectric polymers. Science.

[10] Furukawa T (1989). Ferroelectric properties of vinylidene fluoride copolymers. Phase Transit..

[11] Martins P, Lopes AC, Lanceros-Mendeza S (2014). Electroactive phases of poly(vinylidene fluoride): determination, processing and applications. Prog. Polym. Sci..

[12] Bauer S, Gerhard-Multhaupt R, Sessler GM (2004). Ferroelectrets: soft electroactive foams for transducers. Phys. Today.

[13] Garain S (2015). Self-poled transparent and flexible uv light-emitting cerium complex−PVDF composite: a high-performance nanogenerator. ACS Appl. Mater. Interfaces.

[14] Adhikary P, Garain S, Ram S, Mandal D (2016). Flexible hybrid Eu^3+^ doped P(VDF-HFP) nanocomposite film possess hypersensitive electronic transitions and piezoelectric throughput. J. Polym. Sci. B Polym. Phys..

[15] Ghosh SK (2016). Yb^3+^ assisted self-polarized pvdf based ferroelectretic nanogenerator: a facile strategy of highly efficient mechanical energy harvester fabrication. Nano Energy.

[16] Wei T (2014). Mid-infrared fluorescence, energy transfer process and rate equation analysis in Er^3+^ doped germanate glass. Sci. Rep..

[17] Mandal D, Banerjee HD, Goswami MLN, Acharya HN (2004). Synthesis of Er^3+^ and Er^3+^:Yb^3+^ doped sol–gel derived silica glass and studies on their optical properties. Bull. Mater. Sci..

[18] Zhou J, Liu Q, Feng W, Sun Y, Li F (2015). Upconversion luminescent Materials: advances and applications. Chem. Rev..

[19] Sun L-D, Dong H, Zhang P-Z, Yan C-H (2015). Upconversion of rare earth nanomaterials. Annu. Rev. Phys. Chem..

[20] Tanyi EK, Burton BT, Narimanov EE, Noginov MA (2017). Thermal radiation of Er doped dielectric crystals: Probing the range of applicability of the Kirchhoff’s law. Sci. Rep..

[21] Zhao T (2016). An infrared-driven flexible pyroelectric generator for non-contact energy harvester. Nanoscale.

[22] Sebald G, Guyomar D, Agbossou A (2009). On thermoelectric and pyroelectric energy harvesting. Smart Mater. Struct..

[23] Ghosh SK, Alam MM, Mandal D (2014). The *in situ* formation of platinum nanoparticles and their catalytic role in electroactive phase formation in poly(vinylidene fluoride): a simple preparation of multifunctional poly(vinylidene fluoride) films doped with platinum nanoparticles. RSC Adv..

[24] Mandal D, Henkel K, Schmeisser D (2011). Comment on “preparation and characterization of silver poly(vinylidene fluoride) nanocomposites: formation of piezoelectric polymorph of poly-(vinylidene fluoride)”. J. Phys. Chem. B.

[25] Tamang A (2015). DNA-assisted β-phase nucleation and alignment of molecular dipoles in pvdf film: a realization of self-poled bio-inspired flexible polymer nanogenerator for portable electronic devices. ACS Appl. Mater. Interfaces.

[26] Uwamino Y, Tsuge A, Ishizuka T, Yamatera H (1986). X-ray photoelectron spectroscopy of rare earth halides. Bull. Chem. Soc. Jpn..

[27] Nefedov VI, Salyn YV, Shtemenko AV, Kotelnikova AS (1980). X-ray photoelectron study of trans-influence of the Re–Re multiple bond. Inorg. Chim. Acta.

[28] Kim YJ, Park CR (2002). Analysis of problematic complexing behavior of ferric chloride with *n*, *n*-dimethylformamide using combined techniques of FT-IR, XPS, and TGA/DTG. Inorg. Chem..

[29] Sugama T, KuKacka LE, Carciello N, Hocker NJ (1989). Study of interactions at water-soluble polymer/Ca(OH)_2_ or gibbsite interfaces by XPS. Cement Concrete Res..

[30] Oxtoby DW (2000). Catching crystals at birth. Nature.

[31] Ghosh SK, Sinha TK, Mahanty B, Mandal D (2015). Self-poled efficient flexible ferroelectretic nanogenerator: a new class of piezoelectric energy harvester. Energy Technol..

[32] Mohebbi, A., Mighri, F., Ajji, A. & Rodrigue, D. Cellular polymer ferroelectret: a review on their development and their piezoelectric properties. *Adv*. *Polym*. *Tech*., 21686, 10.1002/adv.21686 (2016).

[33] Tashiro K, Kobayashi M, Tadokoro H (1981). Vibrational spectra and disorder-order transition of poly(vinylidene fluoride) form III. Macromolecules.

[34] Karan SK, Mandal D, Khatua BB (2015). Self-powered flexible Fe-doped RGO/PVDF nanocomposite: an excellent material for a piezoelectric energy harvester. Nanoscale.

[35] Choi Y-Y (2015). Enhancement of local piezoresponse in polymer ferroelectrics via nanoscale control of microstructure. ACS Nano.

[36] Milani A, Castiglioni C, Radice S (2015). Joint experimental and computational investigation of the structural and spectroscopic properties of poly(vinylidene fluoride) polymorphs. J. Phys. Chem. B.

[37] Ghosh SK, Mandal D (2016). High-performance bio-piezoelectric nanogenerator made with fish scale. Appl. Phys. Lett..

[38] Ghosh SK, Mandal D (2016). Efficient natural piezoelectric nanogenerator: electricity generation from fish swim bladder. Nano Energy.

[39] Almond DP, Bowen CR (2004). Anomalous power law dispersions in ac conductivity and permittivity shown to be characteristics of microstructural electrical networks. Phys. Rev. Lett..

[40] Fröhlich, H. *Theory of Dielectrics* (Oxford University Press, London, 1947).

[41] Jana S, Garain S, Sen S, Mandal D (2015). The influence of hydrogen bonding on the dielectric constant and the piezoelectric energy harvesting performance of hydrated metal salt mediated PVDF films. Phys. Chem. Chem. Phys..

[42] Zhu L (2014). Exploring strategies for high dielectric constant and low loss polymer dielectrics. J. Phys. Chem. Lett..

[43] Ghosh SK (2017). Electrospun gelatin nanofiber based self-powered bio-*e*-skin for health care monitoring. Nano Energy.

[44] Zhang Y (2017). Enhanced pyroelectric and piezoelectric properties of pzt with aligned porosity for energy harvesting applications. J. Mater. Chem. A.

[45] Ghosh SK, Mandal D (2017). Bio-assembled, piezoelectric prawn shell made self-powered wearable sensor for noninvasive physiological signal monitoring. Appl. Phys. Lett..

[46] Ghosh SK, Mandal D (2017). Sustainable energy generation from piezoelectric biomaterial for noninvasive physiological signal monitoring. ACS Sustainable Chem. Eng..

[47] Lee JH (2015). Thermally induced strain-coupled highly stretchable and sensitive pyroelectric nanogenerators. Adv. Energy Mater..

[48] Zabek D, Seunarine K, Spacie C, Bowen C (2017). Graphene ink laminate structures on poly(vinylidene difluoride) (pvdf) for pyroelectric thermal energy harvesting and waste heat recovery. ACS Appl. Mater. Interfaces.

[49] Zabek D, Taylor J, Boulbar EL, Bowen CR (2015). Micropatterning of flexible and free standing polyvinylidene difluoride (pvdf) films for enhanced pyroelectric energy transformation. Adv. Energy Mater..

[50] Xie MY, Zabek D, Bowen CR, Abdelmageed M, Arafa M (2016). Wind-driven pyroelectric energy harvesting device. Smart Materials and Structures.

