# Supplementary material for: A hybrid strain and thermal energy harvester based on an infra-red sensitive Er3+ modified poly(vinylidene fluoride) ferroelectret structure
Source: Sci Rep. 2017 Dec 1;7:16703. doi: 10.1038/s41598-017-16822-3 (PMC5711940; doi:10.1038/s41598-017-16822-3)
Supplement: Supplementary file 1 — Supplementary information [file 41598_2017_16822_MOESM1_ESM.pdf]

Supplementary information

A hybrid strain and thermal energy harvester based on an infra-red sensitive Er^{3+} modified poly(vinylidene fluoride) ferroelectret structure

Sujoy Kumar Ghosh,[†] Mengying Xie,[‡] Christopher Rhys Bowen[‡], Philip R. Davies[$], David J. Morgan[$] & Dipankar Mandal[*,†,§]

[†]Organic Nano-Piezoelectric Device Laboratory (ONPDL)

Department of Physics

Jadavpur University

Kolkata 700032, India

[§]Institute of Nano Science and Technology,

Phase-10, Sector-64, Mohali-160062, India

[‡]Department of Mechanical Engineering

University of Bath

Bath, BA2 7AY, UK

[$]Cardiff Catalysis Institute

School of Chemistry

Cardiff University

Cardiff CF10 3AT, United Kingdom

[*] E-mail address: dipankar@phys.jdvu.ac.in, dmandal@inst.ac.in

Tel.: +91 8336017243; fax: +91 33 2413 8917

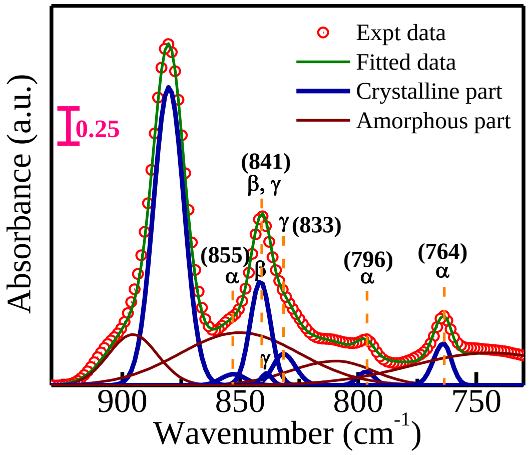

Figure S1. FT-IR spectra of Er-PVDF film in 930−720 cm^{-1} frequency region with deconvolution of the 841 cm^{-1} band to quantify the presence of β- and γ-phases. In order to quantify the individual β-and γ-phases, we have adopted the curve deconvolution technique of the 841 cm^{-1} band where the broadening contribution due to the γ-phase with the sharp and well-resolved peak for the β-phase has been considered. The following equations were used to evaluate the content of isolated β- (equation 1) and γ-phases (equation 2),

$$F(\beta) = F_{EA} \times \left(\frac{A_\beta}{A_\beta + A_\gamma}\right) \times 100\% \qquad \ldots\ldots\ldots\ldots\ldots\ldots\ldots (1),$$

and

$$F(\gamma) = F_{EA} \times \left(\frac{A_\gamma}{A_\beta + A_\gamma}\right) \times 100\% \qquad \ldots\ldots\ldots\ldots\ldots\ldots\ldots (2),$$

where, A_β and A_γ are the integrated areas under the β and γ marked deconvoluted curves.

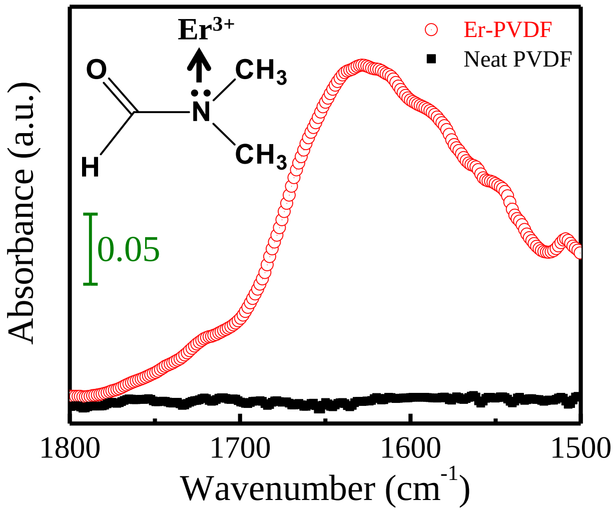

Figure S2. FT-IR spectra of Er-PVDF film in the wavenumber region 1800–1500 cm^{-1} with the coordination complex structure of DMF with the Er^{3+} ion in the inset. This is due to the fact that ligand DMF has two possible coordination sites, *i.e.*, oxygen in the C=O and nitrogen in the C-N bond. According to Gutmann, as a ligand DMF behaves as a strong donor because it exhibits values of donor number (DN) ~ 27.8 and acceptor number (AN) ~ 13.6 which are close to pyridine (DN ~ 24, AN ~ 14.016) that is generally known as one of the best donors (reference 28 in main manuscript). Thus, a coordination complex structure between DMF and Er^{3+} is formed where N coordination site acting as charge donor to Er^{3+}. As a result, strong splitting in N 1s spectra has been observed (Fig. 1d in the main manuscript) due to the two different binding energy states, where, the peak at higher binding energy (~ 402.8 eV) is due to the formation of Er^{3+}–N bond. On the other hand, singlet O 1s spectrum proposes no interaction between O and Er^{3+}. As a result of Er^{3+}–DMF coordination complex, the stretching vibrational band ($\nu_{C=O}$) around 1630 cm^{-1} arises from DMF.

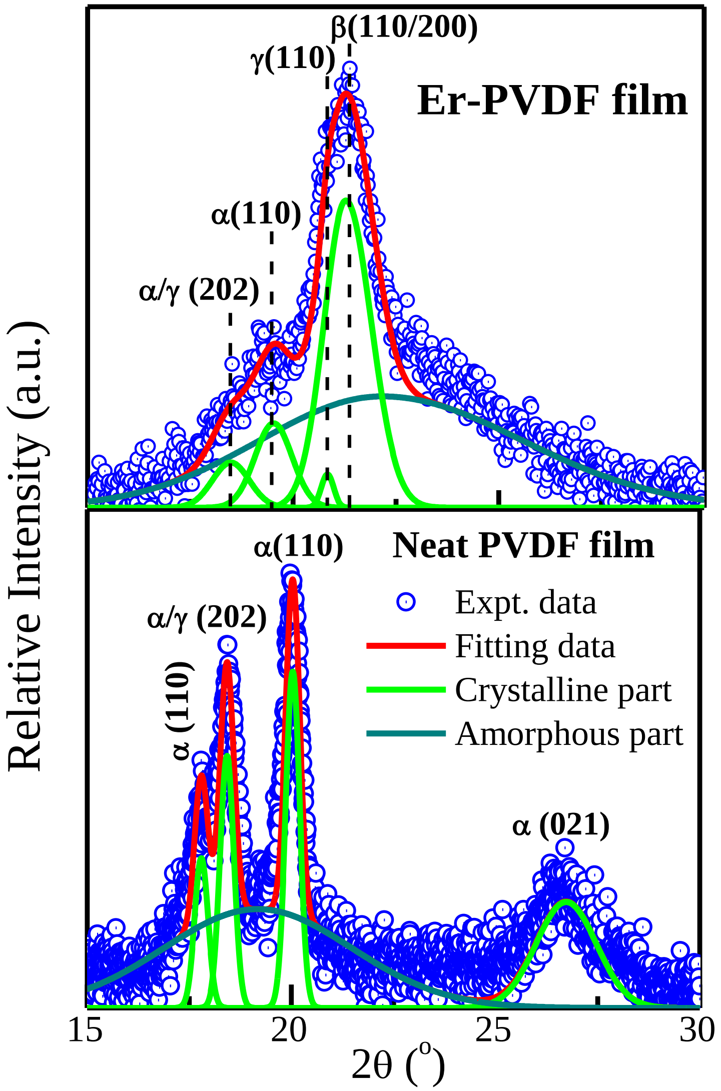

Figure S3. Deconvoluted XRD patterns of Neat PVDF (lower panel) and Er-PVDF (upper panel) films in 2θ ~ 15–30° range. The Neat PVDF film shows characteristic diffraction peaks of α-crystalline phase at 17.6˚(100), 18.4˚(202), 19.9˚(110) and 26.2˚(021) as shown in the FT–IR spectra (Figure 1a). However, the Er–PVDF film exhibits an intense diffraction peak at 20.8° (110/200) which is a signature of the β-phase. An obvious decrease of the crystallinity (χ_c) occurs in the Er-PVDF film ($\chi_c = 38\%$) compared to the Neat PVDF film ($\chi_c = 51\%$) due to

broadening of intense diffraction peak, which was obtained from the curve the deconvolution process. Here, the degree of crystallinity (χ_c) is calculated from

$$\chi_c = \frac{\sum A_{cr}}{\sum A_{cr} + \sum A_{amr}} \times 100\% \quad \ldots\ldots\ldots\ldots\ldots\ldots\ldots\ldots\text{(3)}$$

where, $\sum A_{cr}$ and $\sum A_{amr}$ are the summation of integral area of crystalline peaks and amorphous halo respectively. In addition, the degree of β-crystallinity in the Er-PVDF film was evaluated as,

$$\chi_\beta(\%) = F(\beta) \times \chi_c(\%) = 28\ \% \quad \ldots\ldots\ldots\ldots\ldots\text{(4)}$$

In addition, the crystallite size of β (D_β) crystals in the Er-PVDF film is evaluated from Debye–Scherrer equation as

$$D = \frac{k\lambda}{\beta \cos\theta} \quad \ldots\ldots\ldots\ldots\ldots\ldots\ldots\ldots\ldots\text{(5)}$$

where k is a constant (0.89), λ is the wavelength (0.154178 nm) of the X-ray radiation, β is the full-width (in radian) at half-maximum (FWHM) of the intense diffraction peak, and θ is the angle of diffraction. The large crystallite size (~ 10 nm) assists the molecular (*i.e.*–CH_2 or –CF_2) dipoles to be more co-operative to provide higher ferroelectric responses.

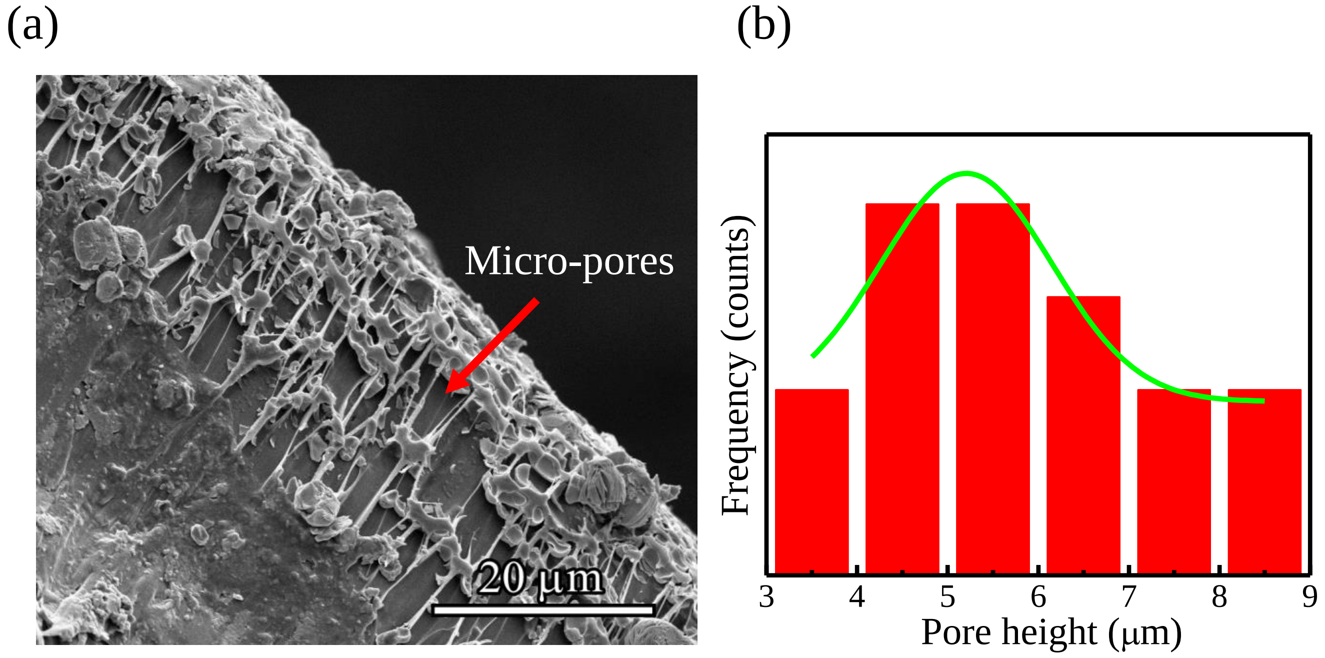

Figure S4. (a) Cross-sectional FE-SEM image of the Er-PVDF film showing non-continuous and layer by layer micro-porous structure along the thickness direction up to (b) the certain average depth of 5 μm shown by the histogram profile of the pore heights ranging from 3.5 μm to 8.5 μm.

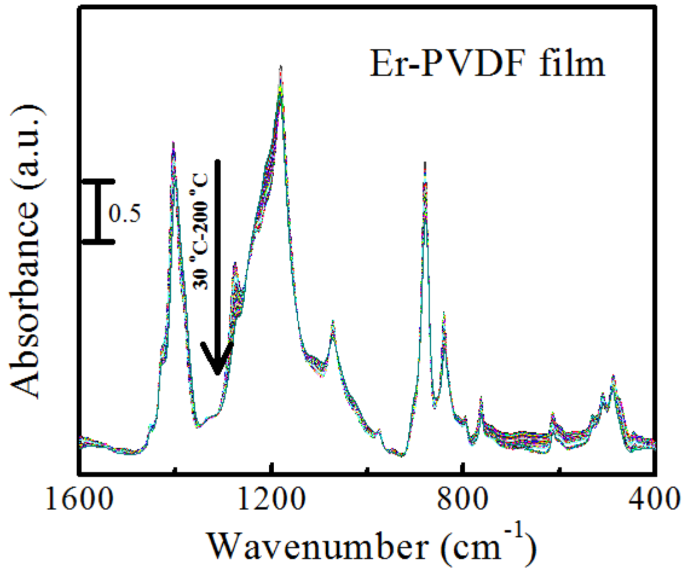

Figure S5. *In-situ* temperature dependent FT-IR spectra of Er-PVDF film, where the spectra between 30°C to 200°C are shown in 1°C intervals, as shown from the indicated vibrational band.

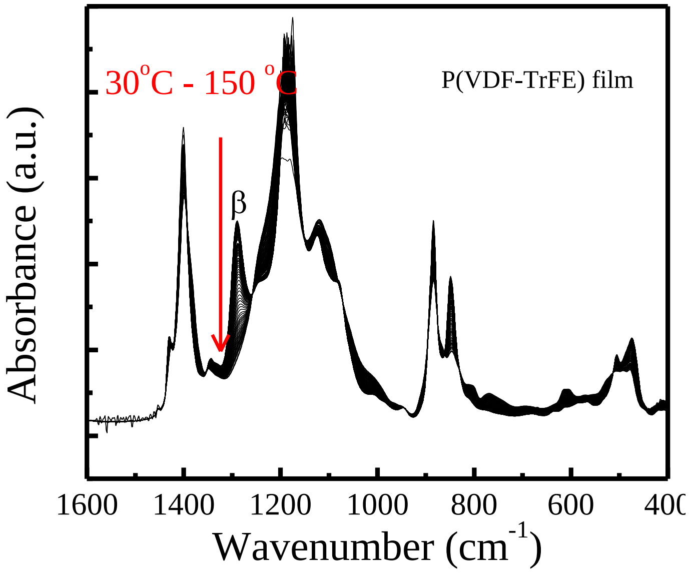

Figure S6. *In-situ* temperature dependent FT-IR spectra of P(VDF-TrFE) film, where the spectra between 30°C to150°C are shown in 1°C intervals, as shown from the indicated vibrational band.

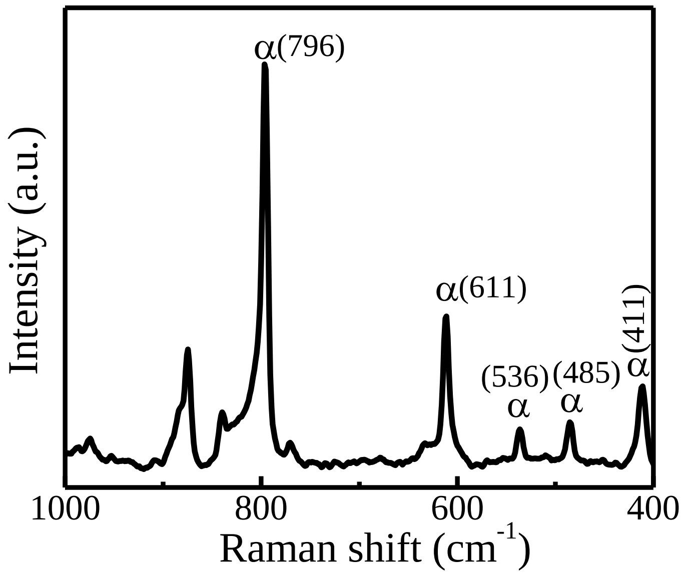

Figure S7. Raman spectra of Neat PVDF film with the bands at 796, 611, 536, 485 and 411 cm^{-1} mainly represent α-phase (reference 36 in the main manuscript).

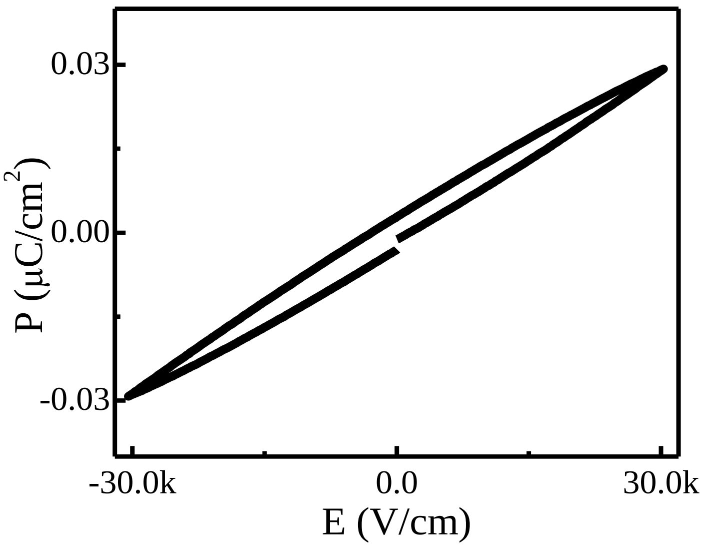

Figure S8. P-E hysteresis loop of the Neat PVDF film showing no switching of the dipoles under the same condition as measured for the Er-PVDF film.

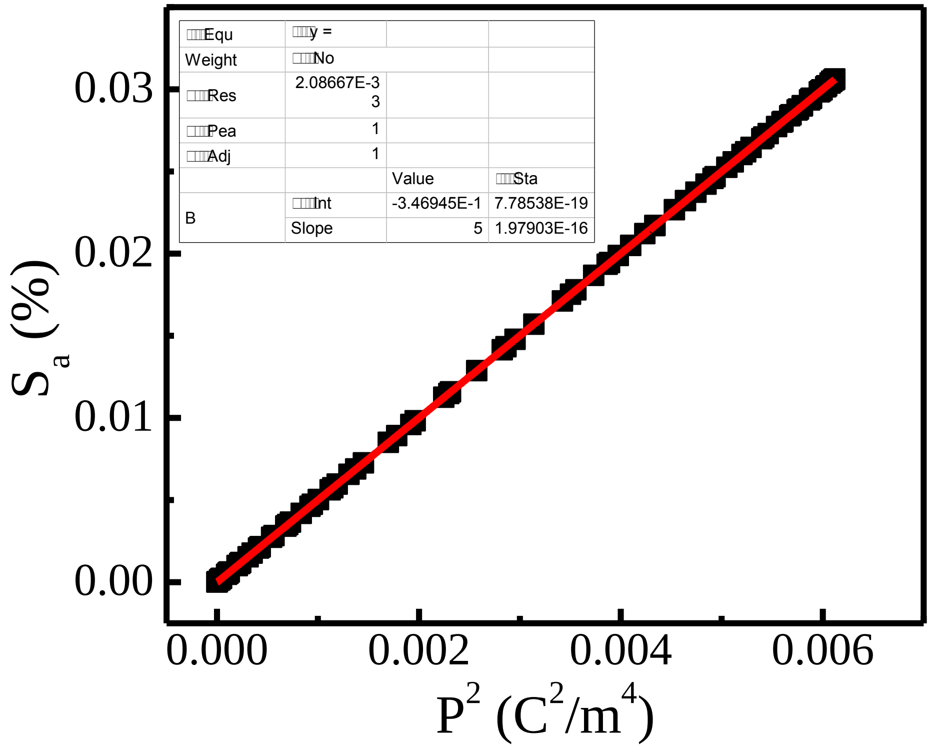

Figure S9. Linear regression of strain (S_a) versus P^2 plot to evaluate the longitudinal electrostrictive coefficient, Q ~ 5 m^4C^{-2} from the slope of the curve according to the equation, $S_a=QP^2$.

Table S1

Output Voltage (V)	Stress, σ_a (Pa)	Strain, $\varepsilon = \sigma_a S_{33}$	Strain rate, $\dot{\boldsymbol{\varepsilon}}$ (% s^{-1}) = $f\varepsilon \times 100\ \%\ s^{-1}$ where f is the frequency
28	0.3×10^6	3.3×10^{-4}	0.165
22	0.2×10^6	2.2×10^{-4}	0.11
18	0.1×10^6	1.1×10^{-4}	0.055
15	10×10^3	1.1×10^{-5}	5.5×10^{-3}
10	1×10^3	1.1×10^{-6}	5.5×10^{-4}
6	100	1.1×10^{-7}	5.5×10^{-5}
4	50	5.5×10^{-8}	2.75×10^{-5}
2	25	2.75×10^{-8}	1.375×10^{-5}
1.2	10	1.1×10^{-8}	5.5×10^{-6}
0.07	1.2	1.32×10^{-9}	6.6×10^{-7}

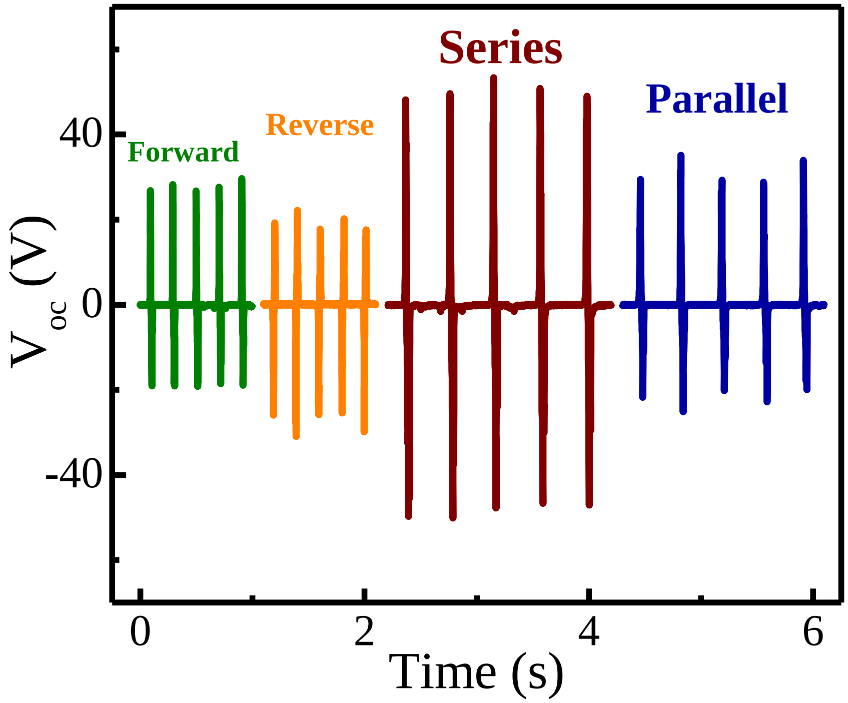

Figure S10. Generated open-circuit voltage (V_{oc}) from single FTNG in forward and reverse connections as well as from two FTNGs when they are connected in series and parallel configurations.

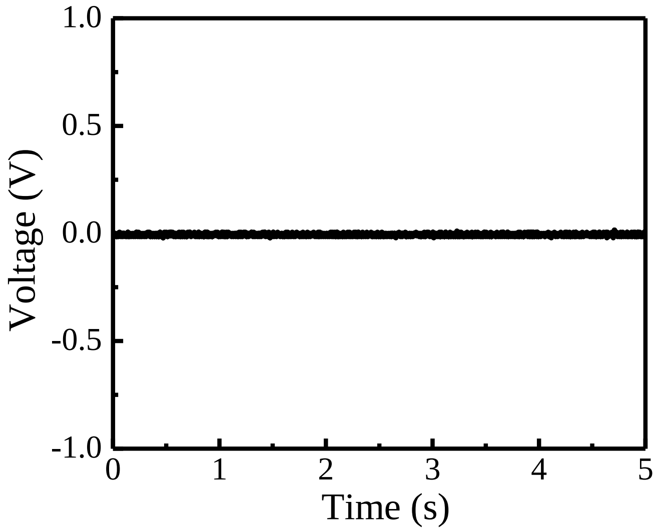

Figure S11. Generated open circuit voltage profile against time from the PDMS based control device without Er-PVDF film which demonstrates no observable output voltage from the device.

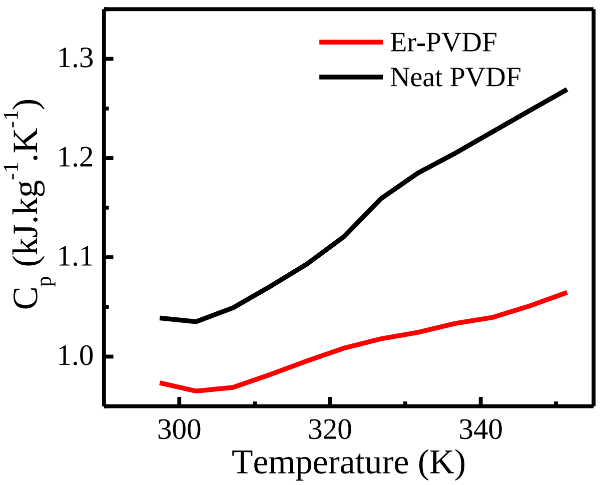

Figure S12. Temperature dependent specific heat capacity (C_p) of Neat PVDF and Er-PVDF films. The $C_{p/332K}$ ~ 1.02 kJ.kg^{-1}K^{-1} has been taken for pyroelectric figure of merit calculation of Er-PVDF as it generates maximum current at this temperature.
